# Polyethylene Glycol and Caspase Inhibitor Emricasan Alleviates Cold Injury in Primary Rat Hepatocytes

**DOI:** 10.21203/rs.3.rs-3669876/v1

**Published:** 2023-11-28

**Authors:** Huyun Chen, Bradley W. Ellis, Antonia T. Dinicu, Mohammadreza Mojoudi, Benjamin T. Wilks, Shannon N. Tessier, Mehmet Toner, Korkut Uygun, Basak E. Uygun

**Affiliations:** Center for Engineering in Medicine and Surgery, Department of Surgery, Massachusetts General Hospital; Center for Engineering in Medicine and Surgery, Department of Surgery, Massachusetts General Hospital; Center for Engineering in Medicine and Surgery, Department of Surgery, Massachusetts General Hospital; Center for Engineering in Medicine and Surgery, Department of Surgery, Massachusetts General Hospital; Center for Engineering in Medicine and Surgery, Department of Surgery, Massachusetts General Hospital; Center for Engineering in Medicine and Surgery, Department of Surgery, Massachusetts General Hospital; Center for Engineering in Medicine and Surgery, Department of Surgery, Massachusetts General Hospital; Center for Engineering in Medicine and Surgery, Department of Surgery, Massachusetts General Hospital; Center for Engineering in Medicine and Surgery, Department of Surgery, Massachusetts General Hospital

**Keywords:** Biological Sciences, Cryobiology, Preservation, Cell Preservation, Organ Preservation, Liver Preservation, Hepatocytes/Hepatology

## Abstract

Current methods of storing explanted donor livers at 4°C in University of Wisconsin (UW) solution result in loss of graft function and ultimately leads to less-than-ideal outcomes post transplantation. Our lab has previously shown that supplementing UW solution with 35-kilodalton polyethylene glycol (PEG) has membrane stabilizing effects for cold stored primary rat hepatocytes in suspension. Expanding on past studies, we here investigate if PEG has the same beneficial effects in an adherent primary rat hepatocyte cold storage model. In addition, we investigated the extent of cold-induced apoptosis through treating cold-stored hepatocytes with pan caspase inhibitor emricasan. In parallel to storage at the current cold storage standard of 4°C, we investigated the effects of lowering the storage temperature to −4°C, at which the storage solution remains ice-free due to the supercooling phenomenon. We show the addition of 5% PEG to the storage medium significantly reduced the release of lactate dehydrogenase (LDH) in plated rat hepatocytes and a combinatorial treatment with emricasan maintains hepatocyte viability and morphology following recovery from cold storage. These results show that cold-stored hepatocytes undergo multiple mechanisms of cold-induced injury and that PEG and emricasan treatment in combination with supercooling may improve cell and organ preservation.

## Introduction

Liver transplantation is a life-saving treatment for end-stage liver disease and liver failure, but the availability of donor organs is limited by factors such as inadequate preservation for extended durations following donor explant [1, 2, 3]. The University of Wisconsin (UW) preservation solution was introduced in the mid-1980s [4, 5, 6], and hypothermic liver preservation by immersion in UW solution on ice or at 4°C, termed static cold storage (SCS), remains widely used today in clinical applications [7, 8]. Nonetheless, prolonged storage is well known to increase the risk of graft dysfunction that contributes to complications post-transplantation [7, 9]. Encouragingly, groups have made considerable progress developing improved preservation protocols to minimize preservation-related injury and prolong storage duration in *ex vivo* whole livers [10, 11, 12, 13, 14, 15]. However, although these studies have increased our knowledge of how specific preservation protocols can lead to greater liver graft outcomes, whole organ studies provide limited options for deeper investigations into mechanisms of cellular survival or damage.

On the cellular level, it has been seen that hepatocytes following SCS display characteristics associated with necrosis and apoptosis, with additional detrimental effects that are not yet well understood [16, 17, 18, 19]. Our lab has previously shown that lipid peroxidation contributes to membrane damage in cold stored suspended hepatocytes, and that UW supplemented with PEG can reduce the degree of lipid peroxidation and stabilize the cellular membrane; the same study also showed the beneficial effects of long-term (> 3 days) storage at a subzero, non-freezing temperature of −4°C, at which the storage solution remains unfrozen due to the supercooling phenomenon [20]. Though, suspended hepatocytes are commonly seen to have decreased attachment efficiency post-preservation, offering limited options to assess cell survivability, function, and morphology over extended culture periods post-storage [20, 21]. In addition, studies have only assessed suspension hepatocytes for viability and function following storage at subzero, nonfreezing temperatures, which may produce different results than plated hepatocytes [20, 21, 22, 23].

Our lab has previously developed a collagen sandwich culture system to mimic hepatocytes’ *in vivo* environment and allow for extended culture (> 7 days) of primary rat hepatocytes [24]. Using a modified version of the collagen sandwich culture system, we evaluated in this study the viability and metabolic function of cultured rat hepatocytes following hypothermic preservation in UW solution at supercooling temperature −4°C, and compared the results obtained with conventional SCS at 4°C. Expanding on prior work, this study aims to investigate if PEG supplementation in UW solution for cold storage at +/−4°C will also provide membrane stabilization for adherent hepatocytes similar to previously explored suspended hepatocytes. In addition, we hypothesize that pan-caspase inhibitor emricasan treatment during recovery can improve cold-stored hepatocyte viability. The hepatocytes were stored for 24-hours, which is the maximum time that rat and human livers have been stored with UW before being deemed unviable for transplantation [6, 10, 25].

## Materials and Methods

### 1. Hepatocyte Isolation

Primary rat hepatocytes were sourced from the Cell Resource Core at Massachusetts General Hospital (MGH) and were used for all experiments. The animals were maintained per National Research Council guidelines, and the isolation was performed in accordance with protocol #2011N000111, as approved by the Institutional Animal Care and Use Committee (IACUC) at Massachusetts General Hospital (MGH). Hepatocytes were isolated from two to three-months-old female Lewis rats (Charles River, USA), as described previously [10]. Experiments were conducted with cells from isolations resulting in a minimum cell viability of 90%, as determined by trypan blue exclusion or acridine orange and propidium iodide (AOPI) staining with the Cellometer K2 (Nexcelom Bioscience, USA). Following isolation, the cells were stored on ice for a maximum of 3 hours in seeding media. The seeding media consists of Dulbecco’s Modified Eagle Medium (DMEM) (Gibco, USA), supplemented with 200 units/mL penicillin and 200 μg/mL streptomycin (Sigma-Aldrich, USA), and 10% fetal bovine serum (FBS) (Gibco, USA).

### 2. Hepatocyte Culture

Plastic 24-well tissue-culture plates were first coated with type I collagen (R&D Systems, USA) at 15 μg/cm^2^. Then, each well was seeded with hepatocytes at a density of 1.25×10^5^ cells/cm^2^ in seeding media. Following one hour of incubation at standard culture conditions (37°C, 5% CO_2_) to allow for cell attachment, the media was changed to culture media. The culture media consists of Williams’ E Medium (Sigma-Aldrich, USA), supplemented with 20 ng/mL epidermal growth factor (EGF) (Sigma-Aldrich, USA), 7.5 μg/mL hydrocortisone (Pfizer Inc., USA), 7 μg/mL glucagon (Boehringer Ingelheim, USA), 0.5 unit/mL insulin (Eli Lilly, USA), 200 units/mL penicillin and 200 μg/mL streptomycin (Sigma-Aldrich, USA), and 10% FBS (Gibco, USA). In culture media, the cells are stabilized overnight at standard culture conditions. The following day, the media was aspirated, and a top gel was prepared in each well by adding 100 μL of type I collagen diluted in sodium bicarbonate-buffered High-Glucose DMEM (Gibco, USA) at a final collagen concentration of 1.25 mg/mL and final pH of 7.4. This solution was incubated at 37°C for one hour to allow for collagen polymerization to complete the top-gel culture setup. Following polymerization, 400 μL of culture media was added to each well and the cells are stabilized overnight for an additional time at standard culture conditions.

### 3. Cold Incubation and Recovery

**Figure 1:**
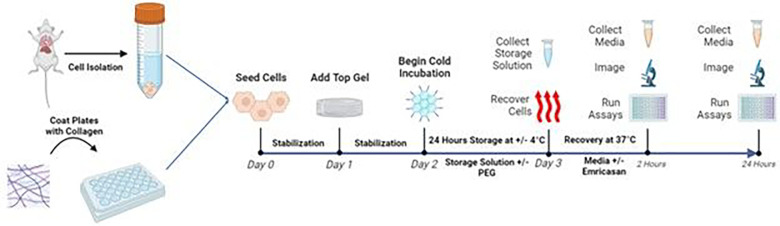
Experimental Design. Schematic illustration of the experimental design and groups. Made with Biorender.

Hepatocytes were stored at two temperatures for 24 hours: 4°C to replicate current static cold storage methods (SCS) or non-freezing subzero −4°C (SZ). The SCS groups were stored in a 4°C fridge while the SZ groups were stored in a portable freezer (Engel-USA Inc., USA). At both storage temperatures, the cells were stored in either UW solution supplemented with 5% 35 k-PEG (Sigma-Aldrich, USA) or plain UW solution (total of four storage groups). Following storage, the storage solution was collected for later analysis and the four storage groups were each rescued with culture media supplemented with or without 5 μM pan-caspase inhibitor emricasan (Selleck Chemicals, USA). The cells were then incubated at standard culture conditions with assessments occurring at 2 and 24 hours after recovery from storage with separate plates being used for each assessment. In addition, a group was only maintained at standard culture without cold storage and assessed on the same day as the cold storage groups. The experimental flow is depicted in Fig. 1.

### 4. Morphology Assessment

Cell staining was performed using a Live/Dead viability kit, consisting of calcein AM and ethidium homodimer stains, and a Hoescht nuclear stain (all from Thermo Fisher Scientific, USA) as per the manufacturers’ protocols. Images were taken with the EVOS M5000 inverted microscope (Thermo Fisher Scientific, USA).

### 5. Cell Metabolism Assay

Resazurin reduction activity was measured by the PrestoBlue assay (Invitrogen, USA). PrestoBlue reagent was added to 37°C culture media at a 1:9 ratio to create a working reagent and 500 μL was added to each well. The cells were then incubated at standard culture conditions for 30 minutes. After 30 minutes, the plate was gently tapped 10 times on the sides to mix, and 300 μL from each well was divided into three wells of a black, flat 96-well plate. The fluorescence was read from the top of the well using the Synergy 2 microplate reader (Biotek, USA) per the manufacturer’s protocol to determine cellular resazurin reduction activity. The plain working reagent was measured to determine the background baseline. Resazurin reduction activity are presented as a percentage of the activity in the SCS no treatment group.

### 6. Lactate Dehydrogenase Release Assay

Membrane integrity was assessed by measuring the amount of cytoplasmic lactate dehydrogenase (LDH) released into the medium, using an LDH detection kit (Sigma-Aldrich, USA) following the manufacturer’s protocol. The absorbance was read from the top of the well using the SpectraMax iD3 microplate reader (Molecular Devices, USA). LDH activity levels are presented as a percentage of the activity in the SCS no treatment group.

### 7. Caspase 3/7 Activity Assay

The caspase 3/7-glo (Promega, USA) assay reagent was prepared beforehand by a 1:1 combination with culture media to create a working reagent and 400 μL was added to each well. The plate was then left on a shaker set at 150 rpm for 30 minutes at room temperature while protected from light. Following 30 minutes, 200 μL from each well was transferred to a white, flat bottom 96-well plate. The luminescence was read from the top of the well using the Synergy 2 microplate reader (Biotek, USA) per the manufacturer’s protocol. The plain working reagent was measured to determine the background baseline. Caspase 3/7 activity levels are presented as a percentage of the activity in the SCS no treatment group.

### 8. Statistical Analysis

Differences in cellular viability markers (LDH, Caspase, Resazurin Reduction) between different temperature and treatment groups were analyzed through a full-effect, two-way analysis of variance (ANOVA). Tukey’s post-hoc test was used to compare the mean of every group with each other within their timepoints (0-, 2-, and 24-hours following recovery). Statistical significance is determined by *p* ≤ 0.05. All analyses were performed in Prism 10 (GraphPad Software Inc., USA).

## Results

### Cold storage for 24-hours at +/− 4°C results in decreased metabolism, membrane damage, and increased caspase activity in hepatocytes.

**Figure 2:**
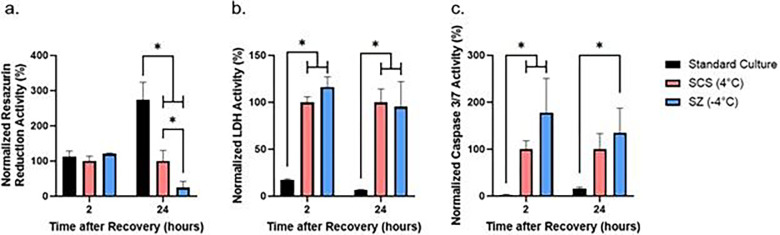
Effect of non-freezing hepatocyte cold storage at +/−4°C on resazurin reduction, LDH, and caspase 3/7 activity. Resazurin reduction (a), LDH (b), and Caspase 3/7 (c) activity is normalized to the activity of the SCS group. * = *p*≤0.05 (n=3 biological replicates)

We tested the effect of 24-hour cold storage on plated rat hepatocytes and observed no change in metabolism between groups at 2-hours following recovery from cold storage (Fig. 2a). Following 24-hours of recovery, we observed significantly lower cell metabolism in both temperature groups as compared to standard culture, with the SZ storage group showing significantly lower metabolism than the SCS group (Fig. 2a). We also observed greater extracellular LDH activity (Fig. 2b) at 2-hours and 24-hours and greater caspase 3/7 activity (Fig. 2c) at 2-hours following recovery from both storage temperatures as compared to the standard culture group. Additionally, we observed greater caspase 3/7 activity at 24-hours in the SZ group when compared to standard culture (Fig. 2C).

### PEG supplementation during storage alleviates membrane damage and increase cell metabolism

**Figure 3:**
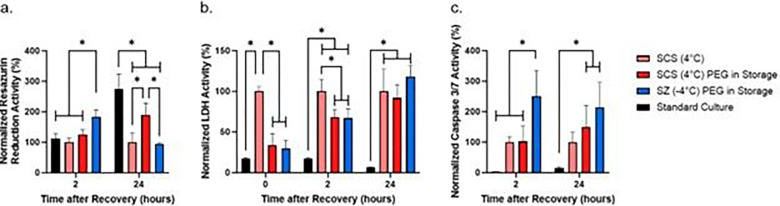
Effect of PEG supplementation during storage on resazurin reduction, LDH, and caspase 3/7 activity. Resazurin reduction (a), LDH (b), and Caspase 3/7 (c) activity is normalized to the activity of the SCS no treatment group. * = *p*≤0.05 (n=3 biological replicates).

We investigated the membrane stabilization effect of PEG during cold storage by supplementing the storage solution with 5% PEG. The general metabolism of cells measured by the PrestoBlue resazurin reduction activity assay following 2 hours of recovery found the SZ-PEG group to have significantly higher metabolic activity than the SCS and standard culture groups (Fig. 3a). Following 24 hours of recovery, all cold storage groups were found to have significantly lower metabolic activity than standard culture (Fig. 3a). Of the cold-stored groups, we observed the SCS-PEG group to have significantly higher metabolic activity than the SCS and the SZ-PEG group (Fig. 3a).

The assessed extracellular LDH activity in both SCS-PEG and SZ-PEG groups are comparable to standard culture immediately after storage, and significantly lower than the activity of the SCS group until 2 hours of recovery (Fig. 3b). However, following 24 hours of recovery, no difference was seen in extracellular LDH activity between the three cold storage groups (Fig. 3b).

We observed the caspase 3/7 activity of the SZ-PEG group following 2 hours of recovery to be significantly higher than the rest of the groups (Fig. 3c). After 24 hours of recovery, caspase 3/7 activity of the SCS-PEG and SZ-PEG groups was measured to be significantly higher than standard culture, as well as higher than the non-treated SCS group although without significant differences (p=0.21 and p=0.10 respectively, Fig. 3c).

### Emricasan supplementation during recovery alleviates caspase 3/7 activity following cold storage

**Figure 4:**
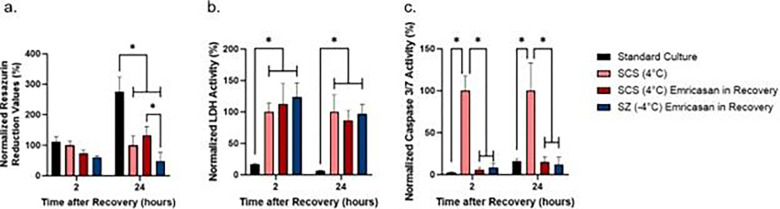
Effect of emricasan treatment during recovery on resazurin reduction, LDH, and caspase 3/7 activity. Resazurin reduction (a), LDH (b), and Caspase 3/7 (c) activity is normalized to the activity of the SCS no treatment group. * = *p*≤0.05 (n=3 biological replicates).

To alleviate the observed increase in caspase 3/7 activity following cold storage as compared to standard culture (Fig. 2c), hepatocytes were recovered with culture media supplemented with 10 μM emricasan, a pan-caspase inhibitor. We observed no difference in metabolic activity between all groups following 2 hours of recovery. Following 24 hours of recovery, all cold storage groups were again seen to have significantly lower metabolic activity than standard culture (Fig. 4a). Following 24 hours of recovery, the metabolic rate in the SZ-emricasan group is significantly lower than its SCS counterpart (Fig. 4a).

In addition, we observed that emricasan has no effect on reducing LDH release in treated groups, with all cold storage groups having greater extracellular LDH activity following both 2 and 24 hours of recovery (Fig. 4b). However, the addition of emricasan in recovery media significantly decreased caspase 3/7 activity in both SZ-emricasan and SCS-emricasan groups following 2 and 24 hours of recovery to levels comparable to standard culture (Fig. 4c). Furthermore, we have observed no difference in caspase 3/7 activity between emricasan treatment in storage solution versus in recovery media only (Supplemental Fig. 2c & 2d).

### Combined treatment of cells with PEG supplementation during storage and emricasan in recovery results in the greatest cell metabolism

**Figure 5:**
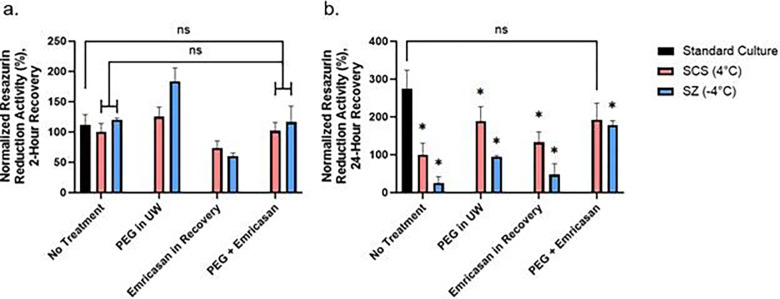
Comparison of resazurin reduction activity between treatment groups. The resazurin reduction activity was determined using PrestoBlue following (a) 2 hours and (b) 24 hours of recovery. The activity is normalized to the activity of the SCS no treatment group. *= significantly different than standard culture (n=3 biological replicates).

Following treatment with PEG and emricasan individually, we wanted to observe the alleviating potential of a combined treatment on preserving metabolic activity by limiting LDH release and inhibiting caspase activity (Supplemental Fig. 1a & 1b). Following treatment with PEG in storage and emricasan in recovery, we measured the metabolic activity of hepatocytes at 2- and 24- hours of recovery for the groups that received no treatment, PEG in UW, emricasan in recovery, and combined treatment. At 2 hours following recovery from storage (Fig. 5a), we did not observe differences between the no treatment groups and corresponding combined treatment groups at both storage temperatures. At 24 hours post-storage, we observed that the metabolic activities of the combined treatment groups are significantly higher than corresponding no treatment groups (Fig. 5b), with the metabolic activity of the SCS-combined treatment group being comparable to standard culture (p=0.52).

### Hepatocyte morphology is preserved following cold storage when the process is supplemented with PEG and emricasan

**Figure 6:**
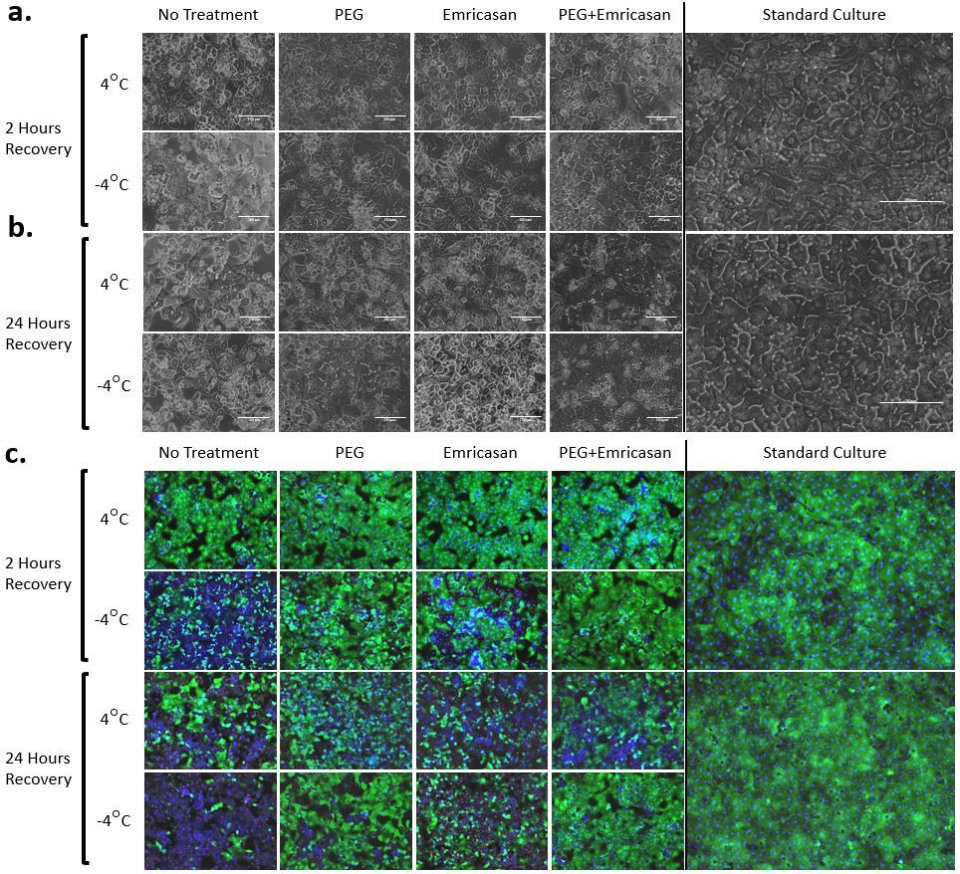
Morphology of Hepatocytes 2- and 24-Hours following Recovery from Storage. Representative phasecontrast microscope images of fresh hepatocytes and cold stored hepatocytes at (a) 2 hours of recovery following cold storage and (b) 24 hours of recovery. 20x magnification. Scale bars: 150 μm. (c) Representative merged fluorescent microscope images of Live (green)/Dead (red) and nuclei (blue) of the cultures at 10x magnification. (n=3 biological replicates).

Phase-contrast and fluorescent microscopy images of hepatocytes were taken following 2 and 24 hours of recovery from cold storage (Fig. 6). Without cold storage, standard hepatocyte cultures displayed an expected polygonal morphology (Fig. 6a & 6b far right images). At 2 hours of recovery following cold storage, significant dysmorphology is noticeable in the SZ group, and to less of an extent in SCS (Fig. 6a). Following 24 hours of recovery, all the non- and partially treated groups for both storage temperatures displayed varying intensities of cell detachment, nuclear collapse, and vacuolization (Fig. 6b). Using the 24-hour SCS no treatment group as an example, although a fraction of dead hepatocytes retains their general shape, their cell membrane is permeable to ethidium homodimer (Fig. 6c). In addition, a fraction of dead hepatocytes was not stained by either ethidium homodimer or Hoescht, suggesting a collapse of the nuclear envelope (Fig. 6c). However, the groups treated with PEG and/or emricasan retained healthy morphology to an extent, with the combined treatment group displaying morphology comparable to the standard culture control (Fig. 6).

## Discussion

Since its conception in the 1980s by Folkert Belzer and James Southard, UW solution for cold storage has increased the quality and duration of preservation for various organs, tissues, and cells [6,7]. With UW solution, whole livers remain transplant viable after up to 24 hours of cold storage [6,10,25]. Though, extension of organ preservation times would enable the establishment of global organ matching networks, allowing for more rigorous donor matches and ultimately lead to greater transplant outcomes [3]. Thus, it is crucial to investigate organ preservation at the cellular level to gain a further understanding of the mechanisms of injury and design improved protocols for better outcomes. In this study, we explored the effects of hypothermic storage at 4°C and −4°C on cultured primary rat hepatocytes and demonstrate that plated hepatocytes cold stored for 24 hours undergo loss of metabolic activity, membrane destabilization, and upregulated caspase 3/7 activity as compared to standard culture.

Our results reinforce the findings of prior studies that similarly observed hypothermic damage in plated hepatocytes following 24 hours of storage in UW solution. Stefanovich *et al*. [26] and Hossein Aghdaie *et al*. [27] both reported that hypothermic treatment in UW solution for 24 hours resulted in significant decrease of albumin secretion. Abrahamse *et al*. [28] observed a 75% increase in LDH release and 60% decrease in metabolic activity from hepatocytes stored in UW solution for 24-hours. In another study, Kerkweg *et al*. [29] reported that 24 hours of hypothermic storage in UW solution or L-15 medium resulted in significant increase of lipid peroxidation and LDH release that can be counteracted by iron chelator supplementation during storage, supporting the commonly observed iron-mediated reactive oxygen species (ROS) generation through the Fenton Reaction [30]. Similarly, Rauen *et al*. [19] has shown that antioxidants can also reduce lipid peroxidation and LDH release, highlighting the role of ROS in hepatocyte cold injury. Though our study did not assess albumin secretion and lipid peroxidation, these studies show the effects of hypothermic-mediated ROS generation on hepatocyte function, which will be an area of focus in future studies.

Previous research has shown that PEG supplementation during hypothermic storage of hepatocytes can also improve survivability [20,25] and reduce lipid peroxidation and LDH release [20,31], supporting the observed antioxidant properties of PEG [32]. Likewise, multiple studies have reported signs of apoptosis in cold-treated hepatocytes [19,28,29,33,34,35]. However, studies did not observe a significant improvement in post-storage viability when only inhibiting caspase activity during cold storage [27,36], while PEG supplementation along with caspase inhibition during storage resulted in significantly higher post-storage viability than cells treated only with PEG during storage [27]. This is consistent with our findings; emricasan treatment during and after storage did not significantly improve metabolic activity and morphology but dual treatment with PEG and emricasan resulted in metabolic activity that is higher than PEG supplementation alone, suggesting contributing effects from multiple death pathways that result in injury following preservation.

Furthermore, we have shown that emricasan during recovery significantly reduces caspase 3/7 activity but did not prevent LDH release, suggesting that caspase inhibition has minimal effect on LDH release following cold storage and that the observed increase in LDH release is not solely caused by apoptosis. This may be explained by the ferroptosis death pathway that is commonly associated with lipid peroxidation [37]. Anegawa *et al*. [38] has recently reported multiple signatures of ferroptosis in cold-treated hepatocytes from mice and hamsters [38], expanding on the findings of previous research that explored the role of iron-mediated ROS generation in hepatocyte cold-injury [29,33,35]. Consequently, our observed increase in LDH release following the replacement of PEG-supplemented storage solution with PEG-free recovery media suggests additional ROS generation during recovery. Following 2-hours of recovery, the metabolic activity of cold-stored hepatocytes is similar to that of the standard culture, but after 24-hours of recovery, standard culture has significantly higher metabolic activity than non-treated cold-stored hepatocytes. This discrepancy suggests that 2-hours of recovery is insufficient for cell stabilization following cold storage and reflects the initially high oxygen consumption commonly seen during the recovery of cold-stored whole livers that is correlated with greater subsequent damage, termed ischemia reperfusion injury and mainly attributed to the generation of reactive oxygen species [14,39,40]. Our results in combination with literature highlight the key role of ROS in hepatocyte injury during both ischemic preservation and the recovery phase following preservation.

Previous research has shown that storing suspended hepatocytes at subzero, non-freezing temperatures improve upon the current cold storage standard [20,21,22,23]. Lower storage temperatures theoretically reduce metabolic activity and cellular processes that could induce cell injury. However, our results show that adherent hepatocytes stored at subzero, non-freezing −4°C leads to lower cell viability than SCS at 4°C following 24-hours of storage. This could be attributed to 24-hours being an insufficient amount of storage time to induce a beneficial effect and that longer storage periods at −4°C may yield different results. It should be considered that cell attachment and interactions mediated by transmembrane proteins may change the characteristics of cell phenotype, such as gene expression and cytoskeletal structure [41], possibly leading to suspended hepatocytes reacting differently to hypothermia than attached hepatocytes and warranting further investigation. In addition, previous research has reported multiple modes of cell injury from different hypothermic storage temperatures in multiple cell lines [42,43], which we hypothesize to also be the case for cultured hepatocytes and will be further explored in future studies.

While we observed no significant differences between the caspase 3/7 activity of non-treated SCS and SZ groups, PEG-supplementation alone resulted in a significant increase in the caspase 3/7 activity between the SCS and SZ groups following 2-hours of recovery, suggesting that non-ferroptotic cells continue caspase upregulation and that additional mechanisms are leading to caspase upregulation with a decrease in storage temperature. Damages to the mitochondria could offer one possible explanation. As the initiator of the intrinsic apoptosis pathway and subsequent caspase activation, the mitochondrion has been commonly observed to undergo a loss in mitochondrial membrane potential and an increased permeability of the inner mitochondrial membrane during ischemia and reperfusion [35,44,45], leading to mitochondria rupture, the release of cytochrome-c, and activation of the caspase cascade. Research has shown iron chelator treatment for inhibiting the loss of mitochondrial membrane potential through the reduction of ROS [35], which are well-known inducers of mitochondria permeability transition [46]. Moreover, research has indirectly shown that ROS generation increases as storage temperature decreases, as observed by temperature dependent increase in LDH release [35] and lipid peroxidation [20]. However, the 35-kilodalton PEG used in this study may be incapable of crossing the plasma membrane due to its size [20,26,47], with evidence suggesting the protective role of PEG is localized at the cell surface [26,47]. These findings collectively suggest that our observed increase in caspase upregulation in PEG-treated cells is due to mitochondrial damage.

Building off the results from this study, our future investigations will aim to elucidate additional death pathways and mitochondrial changes that may also concurrently occur from non-freezing storage at multiple subzero temperatures. It is worth noting that our study only addressed apoptosis and ferroptosis and did not account for other types of hepatic cell death that may also occur [40,42,43,46,48,49,50]. Necroptosis, the regulated form of necrosis in the presence of caspase inhibitors, has been observed during hepatic ischemia-reperfusion through the receptor-interacting protein kinase 3 (RIPK3) pathway [49]. Additionally, Gotoh *et al*. have reported that autophagic death occurs 15 minutes into warm reperfusion of liver grafts, with liver dysfunction attenuated by autophagosome inhibition [50]. Nevertheless, our results from this study in combination with the current literature show that cell survival and death during and following preservation is a multifaceted process with multiple death pathways affecting hepatocyte viability. In future studies, we will continue to investigate the confounding temporal effects caused by different mechanisms of hypothermic injury and further optimize preservation solutions by screening not only for biochemical additives, but also for physical variables such as storage temperature.

In summary, we here show that primary rat hepatocytes experience membrane destabilization following both static cold storage and subzero nonfreezing storage and suffer from apoptosis during recovery. We reinforced previous findings of PEG-supplementation during preservation and introduced pan-caspase inhibitor emricasan as an additional supplement to preserve hepatocyte viability and functionality. The results from this study provide a steppingstone to the development of novel hepatocyte specific preservation solutions for increasing cell health and viability following preservation.

## Data Availability

The datasets generated for the current study are available from the corresponding author upon reasonable request.
